# 3D-Printable Nanocellulose-Based Functional Materials: Fundamentals and Applications

**DOI:** 10.3390/nano11092358

**Published:** 2021-09-11

**Authors:** Abraham Samuel Finny, Oluwatosin Popoola, Silvana Andreescu

**Affiliations:** Department of Chemistry and Biomolecular Science, Clarkson University, Potsdam, New York, NY 13699-5810, USA; finnya@clarkson.edu (A.S.F.); popoolos@clarkson.edu (O.P.)

**Keywords:** nanocellulose, 3D printing, composites, packaging, sustainable materials, additive manufacturing

## Abstract

Nanomaterials obtained from sustainable and natural sources have seen tremendous growth in recent times due to increasing interest in utilizing readily and widely available resources. Nanocellulose materials extracted from renewable biomasses hold great promise for increasing the sustainability of conventional materials in various applications owing to their biocompatibility, mechanical properties, ease of functionalization, and high abundance. Nanocellulose can be used to reinforce mechanical strength, impart antimicrobial activity, provide lighter, biodegradable, and more robust materials for packaging, and produce photochromic and electrochromic devices. While the fabrication and properties of nanocellulose are generally well established, their implementation in novel products and applications requires surface modification, assembly, and manufacturability to enable rapid tooling and scalable production. Additive manufacturing techniques such as 3D printing can improve functionality and enhance the ability to customize products while reducing fabrication time and wastage of materials. This review article provides an overview of nanocellulose as a sustainable material, covering the different properties, preparation methods, printability and strategies to functionalize nanocellulose into 3D-printed constructs. The applications of 3D-printed nanocellulose composites in food, environmental, and energy devices are outlined, and an overview of challenges and opportunities is provided.

## 1. Introduction

**Figure 1:**
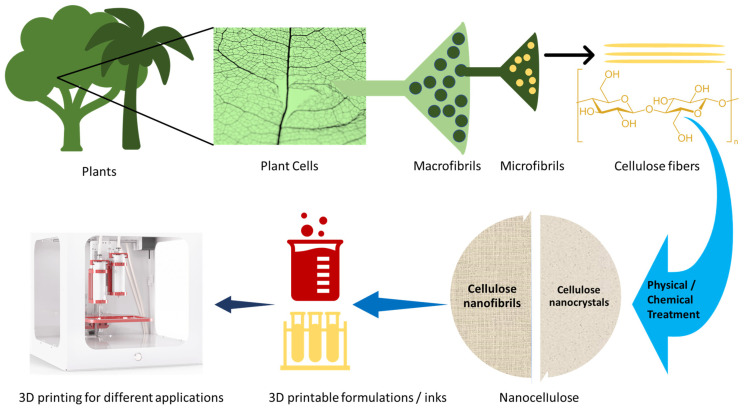
Schematic diagram of nanocellulose: source, processing, and 3D printability.

Nanocellulose, derived from cellulose in the form of cellulose nanocrystals, cellulose nanofibers, or bacterial nanocellulose, has emerged as a robust, new, and versatile material for energy, food, environmental, and biomedical applications [1,2]. Nanocellulose is produced from wood pulp by acid hydrolysis, and the resulting structures have dimensions ranging between 5–100 nm or longer. Its unique properties include a high surface area, high mechanical strength and barrier properties, good biocompatibility, biodegradability, and antimicrobial activity, making it a promising candidate for use in various applications. While the properties of nanocellulose are only beginning to be explored, its promise to replace traditional materials and enable the rapid manufacturing of novel products and applications is high [2]. For nanocellulose to reach its full potential as an alternative sustainable solution to traditional materials, research is needed to develop methods that can convert raw nanocellulose materials into functional constructs made of nanocellulose composites. The latest developments in manufacturing techniques such as additive manufacturing and 3D printing represent a new wave of technology that can help new developments and the realization of these composites and resulting products on an industrial scale. Figure 1 illustrates the transformation of nanocellulose from a raw material derived from plant cells into 3D-printed constructs and the different stages of the transformation process.

Cellulose, poly(1,4-*D*-glucose), is one of the most widespread natural renewable resources on Earth and represents a potential source of high-value products. Cellulose is abundant, can be produced inexpensively on a large scale from sustainable sources, is biocompatible and biodegradable, and can be modified for a variety of applications [3]. Nanocellulose is produced from cellulose by varying chemical and biological processes. Its physical properties, chemical functionality, large surface area, biocompatibility, and biodegradability make nanocellulose a promising material for a broad range of applications. Due to its versatility, nanocellulose can be used as an unlimited source for advanced functional materials, but in order to fully exploit its potential, it is critical to develop strategies to produce derivatives with improved properties in order to expand the range of potential applications. However, nanocellulosic materials do not have sufficient functional properties for advanced applications, and therefore to improve their value and utility, they require modification. Their tailorable functionality can be tuned by selecting surface modifiers or other materials such as natural or synthetic polymers to fabricate composite functional structures and useable products.

The intrinsic properties, size, and composition of native cellulose can be modified by chemical (e.g., solvent) or thermal treatment or by creating hybrid structures in conjunction with other natural or synthetic polymers and biomolecules including epoxy, starch, polyurethane, polylactic acid, hydroxyethyl cellulose, cellulose acetate butyrate, melamine-formaldehyde resins, chitosan, polystyrene, polypyrrole, methacrylates, polyacrylamide, polyacrylate, polyaniline, polyfuryl alcohol, and polyvinyl alcohol [4,5]. The properties of the resulting cellulose-based nanocomposites depend on the structural features of the starting cellulosic materials, which vary heavily on the properties of the cellulose fiber network rather than the individual nanofibers. The bonding and adhesion between the fibers are dependent on the homogeneity and origin of the nanocellulose matrix. To improve the homogeneity of nanocellulose composites, surface functionalization by introducing stable negative or positive electrostatic charges has been used to tweak the surface energy characteristics and graft functional molecules by in situ modification within the nanocellulose matrix [6]. Many hydroxyl groups available on the surface make functionalization possible while maintaining the hydrophobicity of the surface and keeping the composites thermodynamically stable [7].

There are multiple ways in which cellulosic nanocomposites can be produced. Standard methods include casting, electrospinning, melt extrusion, molding, milling, and precipitation. The recent introduction of 2D and 3D printing techniques provides an avenue for scalable and reproducible manufacturing of new materials and devices. These additive manufacturing techniques enable the production of constructs with tailorable structures and enhanced mechanical and chemical properties. Using 3D printing techniques, mechanically stable constructs are fabricated through computer-controlled deposition of materials, layer by layer, until structures with precise macroscopic and microscopic control are created [8]. Increased usage of extrusion-based 3D printing has been seen, which involves the utilization of hydrogels or printable pastes constructed by incorporating viscous materials that can retain their shape after deposition through chemical, photo, or thermal crosslinking methods [9,10]. Nanocellulose can be introduced to these pastes to facilitate the deposition and realization of stable constructs for applications. The selection of materials and their rheological properties decide whether these could be 3D printed and incorporated into applications.

**Figure 2:**
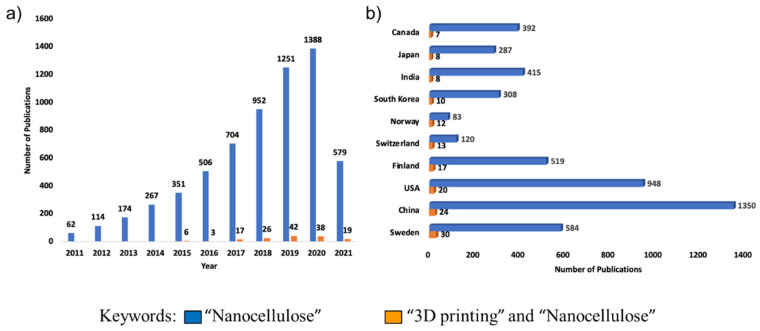
Graphs showing the number of publications between 2011–2021 (**a**) and publications per country (**b**) using Web of Science (accessed on 30 June 2021).

The interest in nanocellulose research has grown substantially in recent years as evidenced by the exponential growth in the number of publications, with the highest numbers from countries such as China, USA, Sweden, and Canada, who have invested heavily in efforts to support reutilization and repurposing of cellulosic materials to promote the development of high-value wood-based products (Figure 2). In the past four years, with the recent growing interest in additive manufacturing, there is also increased use of 3D printing for nanocellulose research. This review article summarizes the potential of nanocellulose as a promising material for the fabrication of functional nanostructures and devices fabricated by 3D printing. Some of the commonly used bioprinters are the EnvisionTec 3D-Bioplotter, RegenHu 3DDiscovery Evolution, Poietis NGB-R and NGB-C 3D bioprinters, Cellink Bio X6, 3D Systems Allevi Series, Rokit Dr. Invivo 4D, Inventia Rastrum, Organovo NovoGen MMX, Aspect Biosystems RX1, and Bioprinting Solutions Fabion 2. Even though most of these printers are used for printing functional tissues and organs, in recent times they have seen a tremendous increase for use in other areas for creating functional materials (Figure 2). The properties, modification, and fabrication of nanocellulose-based nanocomposites and printable inks for food, energy, and environmental applications have seen increased interest in recent times.

## 2. Nanocellulose: Preparation, Treatment, Functionality, and 3D Printability

Nanocellulosic materials include three types of structures [7]: (i) bacterial nanocellulose (BNC), bacterial cellulose (BC), or microbial cellulose; (ii) nanofibrillated cellulose (NFC), also called cellulose nanofiber (CNF) or cellulose nanofibrils; and (iii) nanocrystalline cellulose (NCC), also known as crystallites, whiskers, or rod-like cellulose microcrystals as well as crystalline nanocellulose [11,12,13]. Nanocellulose is considered non-toxic, renewable, biodegradable, and safe [1,14]. Its high aspect ratio gives good strength and toughness as a reinforcement matrix for various formulations in additive manufacturing, food packaging, medical, flexible electronics, military, and energy applications [15,16,17,18,19]. This section discusses the main functional properties of nanocellulose, modification techniques, and composites to create 3D-printable structures.

### 2.1. Preparation

Cellulose is reported to be the most abundant polysaccharide in nature [20]. It is widely distributed as it constitutes the major component of a plant’s cell wall; hence, cellulose can be easily sourced from plants, bacterial pellicles, and agricultural wastes [21,22]. The diameter of BC ranges between 20–100 nm, and it is commonly produced from low-molecular-weight sugars and alcohols. Glucose chains are formed inside the bacterial body during the biosynthesis process and extrude out through tiny pores present on the cell envelope [23]. These chains combine to form microfibrils further aggregated into ribbons to generate a web-shaped network structure with BC [23]. Stefan-Ovidiu D. et al. also reported BNC production from a kombucha membrane obtained as a secondary product from the fermentation of tea broth in beverage production [24]. Several processing methods have been used to produce CNC and CNF from cellulose sources, usually agricultural wastes, by exposing the basic building blocks of cellulose [20]. These methods can be summarized into two: (i) pretreatment or purification of the agricultural biomass for fractionation and (ii) treatment with the isolation of nanocellulose from purified cellulose [25]. The plant cell wall contains lignin and hemicellulose, which serve as a protective coating around the cellulose fibers, and this enables the cell wall to maintain its structured form. To extract the cellulose to produce nanocellulose, a hybrid preparation system is usually employed to remove the protective coating through mechanical forces followed by chemical pretreatment [22]. The chemical pretreatment step involves using chemicals such as alkali, the most common method in the industrial production of cellulose, which enables the effective delignification and partial solvation of the hemicellulose. Common alkalis used for this purpose include NaOH, KOH, and NH_4_OH [26,27].

Other chemical pretreatment methods include oxidative delignification, which involves converting lignin copolymer into carboxylic acids using strong oxidizers such as hydrogen peroxide or ozone. To further ease the penetration of hydrolytic media, oxidative delignification could be followed by effective enzymatic hydrolysis. Due to the low cost of hydrogen peroxide and its effectiveness in delignification pretreatment, the method is widely used on woody biomass. Several combined pretreatment steps are often needed to achieve nanosized materials with a high aspect ratio and crystallinity index [26,27]. Khan et al. produced CNC with an aspect ratio of 10.45 ± 3.44 nm and a crystallinity index of 66.7% by pretreating dunchi fiber with alkali, hydrogen peroxide, and sulfuric acid sequentially [28].

Other works have highlighted the use of ionic liquids (ILs) as a selective pretreatment method for removing the lignin and hemicellulose (soluble) from lignocellulose biomass. The method enables precipitation of the insoluble cellulose, which can then be filtered off and washed. ILs are expensive and have been found to inactivate cellulase enzymes irreversibly; hence it may not be suitable for pretreating cellulose for biological uses [29]. Deep eutectic solvents (DESs) are recently emerging solvents with promising prospects in lignocellulose biomass pretreatment. DESs have similar physicochemical characteristics as ILs and can be prepared at a lesser cost. While ILs are produced from the combinations of particular types of discrete anions and cations, DESs are formed by mixing Bronsted or Lewis acid and bases made up of diverse cationic and anionic species [30], and their physicochemical properties can be tuned to a specific need by adjusting the molar ratio of each constituent [31].

Following the isolation of the nanosized cellulose particles from the pretreated fiber, a sequential chemical, enzymatic, or mechanical treatment is applied. The selection of suitable nanocellulose isolation methods depends primarily on the desired degree of crystallinity and the type of nanocellulose (CNF or CNC) of interest [25]. Fan B. et al. reported the preparation of nanocellulose from the bamboo leaf that was initially pretreated by grinding, sieving, dispersion in water, ultrasonication, oven-drying, functionalization, and then subjected to further mechanical ultrasonication treatment [32]. Fortunati, E. et al. obtained nanofiber with about 10 nm diameter and 175 nm length from a sunflower using sulfuric acid treatment [33]. CNF of ~100 nm and 67% crystallinity was derived through acid hydrolysis of pretreated ginger tuber by Abral H. et al.; however, the crystallinity dropped to 48% after ultrasonication. A common technique is the direct acid hydrolysis of microcrystalline cellulose (MCC), microfibrillated cellulose (MFC), nanofibrillated cellulose (NFC), wood fiber (WF), or plant fiber (PF) to produce highly crystalline needle-like particles with a high aspect ratio of about 10 nm width and several 100 nm in length and made of about 100% cellulose with a high fraction of Iβ crystal structure [1]. A pure form of BC can be synthesized using bacteria (such as *Acetobacter xylinum*) without pretreatment to remove unwanted contaminants or impurities such as hemicellulose, pectin, and lignin [34].

### 2.2. Functional Nanocellulose-Based Composite Nanostructures

Nanostructured materials with defined structures (size, shape, and connectivity) and controllable physicochemical properties (sorption and separation) are of great interest in materials science, opening new avenues for designing functional materials and devices [34,35,36,37,38]. Their advantages include a high surface-to-volume ratio, large pore volume, and the possibility to control charge, accessibility, and availability of surface functional groups. Changes in these properties, i.e., volume, porosity, and shape, can be triggered by varying environmental conditions, pH, ionic strength, water, electricity, or light [39]. This has allowed the development of stimuli-responsive materials with reversible and programmable actuation and can offer promising capabilities in sensing, artificial muscles, and electronic devices [40].

The combination of nanocellulose with other materials, polymers, and fillers can increase mechanical and thermal stability and change the wetting behavior of the native material [41]. For example, significant enhancement of cellulose tensile strength was achieved by loading cellulose with only 0.2% graphene [42]. The ability of nanocellulose to enhance mechanical properties has enabled their use for the fabrication of printable electronics [43] or sustainable food packaging, as a new type of packaging material [44]. Other possibilities include using polymers to change the surface properties, porosity, and wetting characteristics. Water permeation and salt rejection capabilities have been achieved by interfacial polymerization of amino-functional piperazine and 1,3,5-trimesolyl chloride on cellulose, which enabled applications in water purification [45]. Ultrathin nanocellulose shell microparticles were obtained via emulsion-templated colloidal assembly. This method enabled the loading of pH-responsive compounds, such as methylene blue, using polystyrene as a matrix. The carboxyl groups of the CNF shell showed pH-dependent properties and strong adsorption and drug release behavior [46]. Chemical or biological receptors can also be integrated to provide selectivity for sensing applications. In this case, fabrication requires appropriate surface chemistry suitable for the assembly of biomolecules to maintain bioactivity for selective binding.

### 2.3. Three-Dimensional Printability of Nanocellulose Composites

Three-dimensional printing is an emerging technology that applies rapid prototyping techniques to fabricate computer-designed models and integrate complex structures with an unprecedented level of functionality for applications. The method is currently being explored for various materials and devices, and has proven helpful for patterning and assembling nanomaterials in geometrically complex functional structures [47]. The method provides unique opportunities for the large-scale manufacturing of nanocellulose-based constructs and nanocomposite structures. However, achieving the required level of functionality and integration is challenging as it requires the ability to pattern and develop nanocellulose-based inks of suitable rheology, composition, deposition, and crosslinking conditions for realizing printability. Unlike conventional plastics, nanocellulose is typically processed in aqueous solutions, which are used to create printable inks but might result in anisotropic deformation and poor mechanical properties [48]. Therefore, achieving high consistency and ensuring printability with shape retention and structure by 3D printing is challenging. Nevertheless, the integration of nanocellulose with 3D printing can enable the creation of functional structures with optical, mechanical, electrical, and biological properties for many applications. In contrast with conventional deposition methods, 3D printing enables rapid prototyping and provides mass production capabilities, reducing development time, fabrication steps, and cost.

**Figure 3:**
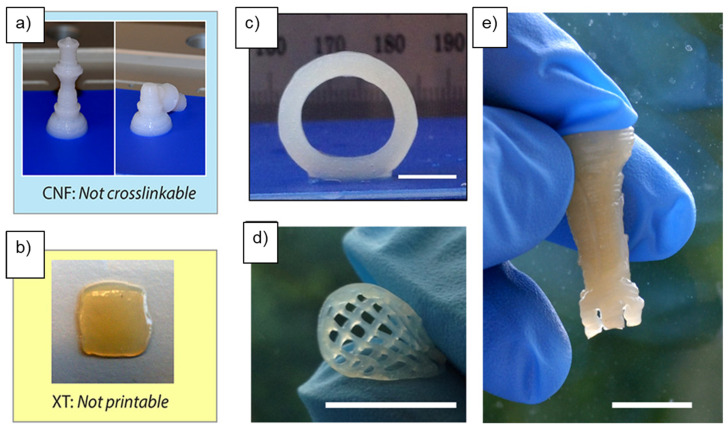
A queen chess piece printed with pure CNFs which collapsed due to lack of crosslinking (**a**), poor printability shown by unsuccessful grid printed with pure XT (**b**). Three-dimensional printed and crosslinked, freestanding and crosslinked cylinder (**c**) grid, handled and bent in air (**d**). Rook chess piece, held upside down from optimized formulation of CNF and XT (**e**), reprinted with permission from ref. [49]. Copyright 2017, American Chemical Society.

Printing of 3D nanocellulose-based devices requires incorporating additional materials, typically polymers and solvents, in conjunction with nanocellulose. The printing process also involves post-processing to consolidate the 3D structure and provide shape consistency. In fabricating composite structures, the size, shape, and type of nanocellulose materials as well as the type and properties of the additional binders and their complementarity play a critical role. It has been shown that when a neat CNF ink is 3D printed into a specific shape, the printed structure collapses when handled or exposed to mechanical force due to the inability of the fibers to crosslink; hence, neat CNF ink is not conducive for 3D printing [49]. Crosslinking enables the 3D-printed material to maintain its shape and mechanical structure. To achieve improved surface properties that enhance structural sustenance, supportive chemical species can be grafted to the reactive end (-OH side groups) of the nanocellulose [1]. Several components have been combined with CNF at varying concentrations, such as biodegradable polymers and hydrogels. Reagents such as xylan-tyramine (XT) [38], which alone is not printable, can be used in a composite structure with CNF at the appropriate concentration to impact crosslinking ability (Figure 3). Silver nanoparticles (AgNPs), a broad-spectrum antimicrobial agent, have been widely used to impact the antimicrobial properties of nanocellulose and fabricate antimicrobial constructs by 3D printing [50,51,52].

**Figure 4:**
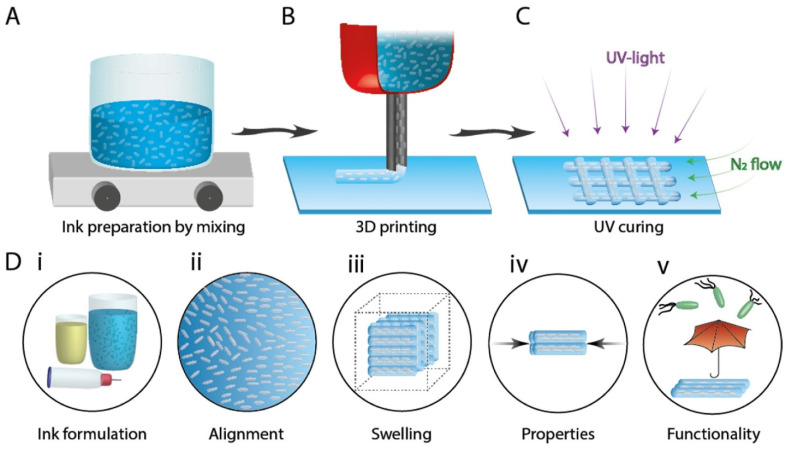
Illustration of the steps involved in the preparation of 3D-printed functional CNC-based hydrogels, involving: (**A**) ink formulation, (**B**) ink writing of cellulose-based polymer ink, (**C**) post-treatment to cure the printed construct, and (**D**) (**i**–**v**) characteristics of inks and printed constructs, reprinted from ref. [53].

In a recent example, the manufacturing of stable structures with cellulose nanocrystals (CNCs) involved: (1) obtaining homogenous dispersion of CNC hydrogel ink by mixing, (2) printing cellulose scaffolds by aligning the anisotropic CNC upon printing, and (3) UV curing of the printed scaffold (Figure 4) [53]. CNC was used in this example as a reinforcing agent (up to 35%), while CNF was added at a lower concentration (1%) to enhance shape retention. N-isopropyl acrylamide (NIPAM), a photo-crosslinkable polymer, was used to produce biocompatible hydrogel inks. The addition of ε-polylysine imparted antimicrobial properties for potential biomedical uses. This composition enabled the ability to control the composite structures’ local orientation, mechanical properties, actuation, and printability while providing additional functionality, e.g., antimicrobial activity and the ability to bend and twist upon hydration. The nanocellulose–NIPAM hydrogels were printed using a direct ink writing procedure with the hydrogels placed in plastic cartridges and extruded through steel nozzles under compressed air. Curing was achieved under UV exposure conditions. It was observed that nanocellulose was able to constrain the swelling and shrinkage of the PNIPAM structures, enabling the production of shape-morphing nanocellulose-based composites through direct ink writing [53].

**Figure 5:**
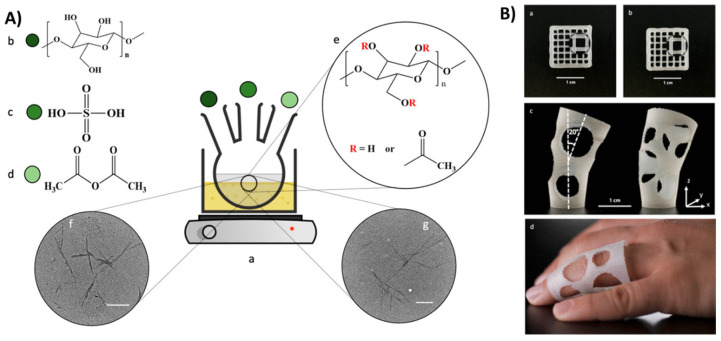
Illustration of CNC acetylation process and 3D-printed nanocellulose–PHBH-based composites showing the (**A**) acetylation process (**a**) of a CNC particle of a cellulose structure (**b**) by sulphuric acid (**c**) and acetic anhydride (**d**), and acetylated nanocellulose after functionalization (**e**–**g**). (**B**) FDM 3D-printed nanocellulose composites of PHBH-acetylated CNC (10%) showing an alteration of 0–90° (**a**–**c**) as an example of a medical device for finger dislocation (**c**,**d**), used with permission from ref. [54].

In another report, a 3D-printed biobased composite made of acetylated CNCs (5–20 wt%) and poly(3-hydroxybutyrate-co-3hydroxyhexanoate) (PHBH), a biodegradable aliphatic polyester was fabricated as an environmentally friendly alternative bioplastic [54]. The method involved the use of a melt-compounding process, preceded by solvent mixing to obtained biobased composites with good dispersion behavior of PHBH and CNCs. An extrusion-based printing, fused deposition modeling (FDM) was further used to print the composite. Acetylation of the CNC was first required to create compatibility between the CNCs and the PHBH and enable the formation of a stable structure (Figure 5).

## 3. Applications of 3D-Printed Nanocellulose-Based Materials

**Figure 6:**
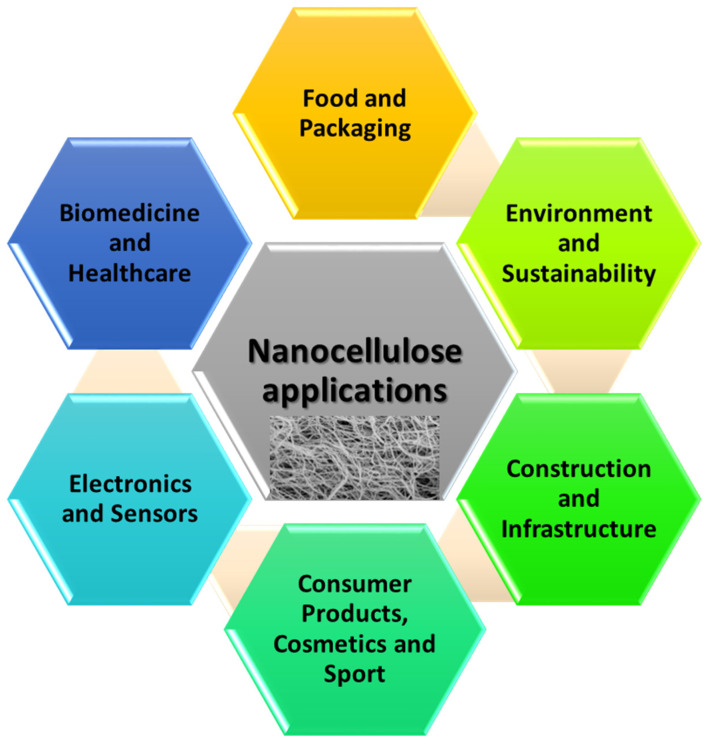
Summary highlighting the broad examples of applications of 3D-printed nanocellulose constructs.

Even though 3D-printable nanocellulose-based composites are still in their infancy, there has been an increase in their applications in different fields ranging from biomedicine, including wound dressing, drug release, and tissue engineering, sensors, food, and packaging, to energy storage and electronics, with growing interest in other areas as well [17,18,19,20,21,22,23,24,25,26,27,28,29,30,31,32,33,34,35,36,37,38,39,40,41,42,43,44,45,46,47,48,49,50,51,52,53,54,55], summarized in Figure 6. This section discusses the recent development in 3D-printed nanocellulose-based composites for food, environmental, food packaging, energy, and electrochemical applications.

### 3.1. Environmental Applications

**Figure 7:**
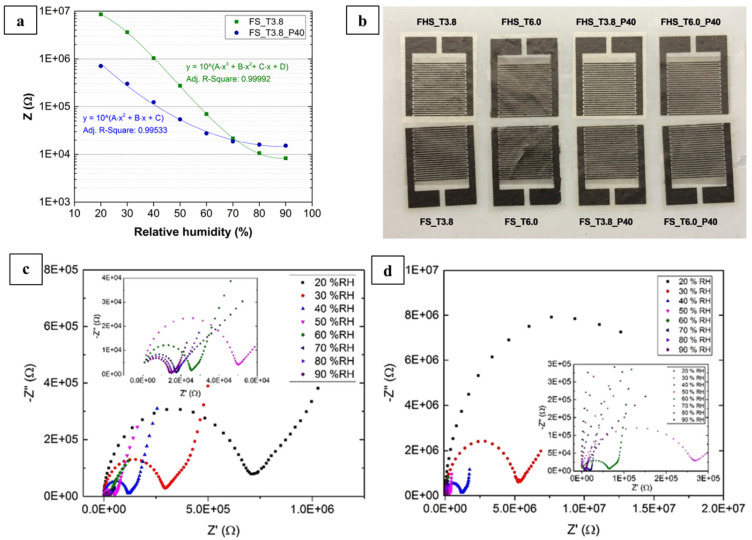
Calibration curves of tested samples (green, without plasticizer, and blue, with plasticizer) (**a**); RH sensors based on CNF films with screen-printed carbon electrodes (**b**). Impedance spectra under different humidity levels for the sample with plasticizer (**c**) and without plasticizer (**d**) at 25  °C. Insets: spectra for humidity levels 50, 60, 70, 80, and 90%, used with permission from ref. [53].

Due to its sustainability, biocompatibility, and ability to form high mechanically robust and stable structures with a broad range of functionalities, nanocellulose has found various applications in the environmental field. Nanocellulose-based printed devices can be used as filters and membranes for water purification and environmental remediation, e.g., for pollutant removal, filtration, and desalination [18], or as materials to create sensors for environmental detection. A printed sensor on a bagasse-derived CNF-based biocomposite was demonstrated to be applicable for a wide range of humidity sensing (20–90% relative humidity, RH) with potential for soil and agricultural products monitoring in the smart farming sector [56]. Bagasse, a dry pulpy residue from the sugarcane industry, was oxidized using a 2,2,6,6-Tetramethylpiperidinyl-1-oxyl (TEMPO)-mediated oxidation process after two different pretreatment methods for comparison, (i) soda and (ii) hot water and soda, to obtain CNF. Four CNF-based sensors were then fabricated by screen printing carbon-based interdigitated electrodes (IDEs), which are reliably sensitive to impedance changes. These four sensors showed decreasing impedance with increasing RH in a humidity chamber experiment with no significant difference in sensing behavior between the four sensors. Polyethylene glycol (PEG) has been reported to enhance the mechanical properties of CNF films and the printability of CNF substrates [57,58]. To enhance the ductility of the CNF film, PEG was used to fabricate self-standing humidity sensors. The response of the sensors containing PEG was fast and stable at each level within the tested range of 7 × 10^5^ Ω (at 20% RH) to 1.5 × 10^4^ Ω (at 90% RH), while the counterpart without PEG, FS_T3.8, covered a more comprehensive range than was represented by the change of impedance level from 8.6 × 10^6^ Ω (at 20% RH) to 8.3 × 10^3^ Ω (at 90% RH), and the average values of the impedances for each RH level were used to construct the calibration curve (Figure 7). However, the incorporation of the PEG plasticizer into the biocomposite film decreased the oxygen barrier significantly.

**Figure 8:**
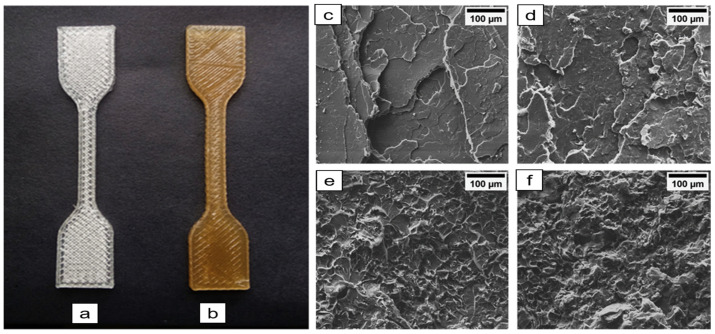
Three-dimensional-printed tensile specimen of PLA (**a**) and PLA/1CNF (**b**) composites, scanning electron microscopy micrographs (×600 magnification) of the tensile fractured surface of compression-molded PLA (**c**), PLA/1% CNF (**d**), PLA/3CNF (**e**), and PLA/5CNF (**f**) composites, used with permission from ref. [59].

**Figure 9:**
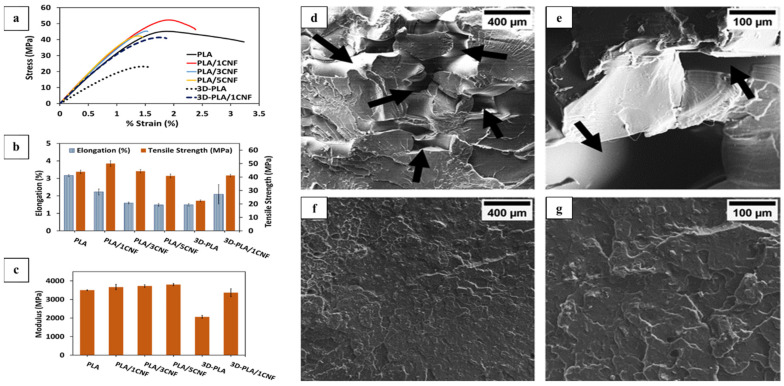
Tensile stress–strain curve of compression-molded and 3D-printed PLA and PLA/CNF composites (**a**) and histograms of mechanical properties of the samples (**b**,**c**). Scanning electron microscopy micrographs (×150 and ×600) of the tensile fracture surface of 3D-printed PLA (**d**,**e**) and 3D-PLA/1% cellulose nanofiber composites (**f**,**g**), used with permission from ref. [59].

Another 3D-printable nanocellulose-enhanced composite of PLA/CNF (polylactic acid/cellulose nanofibers) was successfully printed (Figure 8) by T. Ambone et al., with high tensile strength, stress, and strain. This structure has potential as an eco-friendly packaging material, being fabricated from bioderived and biodegradable matrixes and fillers [59]. PLA/CNF composites were formulated by incorporating 1/3/5 wt% of CNF into the matrix of PLA to improve the poor mechanical properties of PLA and were printed through fused filament fabrication (FFF)-3D printing process. The micrographs of the printed materials from scanning electron microscopy reveal that 3% CNF in PLA was evenly distributed because no CNF aggregates were observed in the fracture surfaces. Furthermore, as indicated with black arrows, the voids, welding points, and overlapped structure (Figure 9) that were responsible for the porosity and lower mechanical properties of the fracture surface morphology of the 3D-printed neat PLA are absent in the fracture surface of PLA/3% CNF nanocomposite, while micro aggregates were seen on PLA/5%CNF. Furthermore, through differential scanning calorimetry, it was discovered that the incorporation of CNF accelerates the nucleation and enhances the crystallinity of the 3D-printed PLA. Further characterization of the PLA/CNF FFF-3D-printed material using X-ray microtomography revealed that the incorporation of the nanocellulose did not compromise the construct’s thermal stability, but it did have lesser voids compared to the ordinary 3D-printed PLA.

Another potential growth area in the environmental field is to use 3D printing to fabricate nanocellulose-based filters and membranes for environmental remediation and cleanup. Due to its high adsorption capacity, functionality, and high specific surface area, nanocellulose is a promising sustainable adsorbent for removing chemical contaminants such as heavy metals and organic dyes. The abundance of the functional group enables further enhancement of the adsorption efficiency, making nanocellulose a particularly suitable sorbent for environmental decontamination and a possible substitute to the currently used adsorbents such as activated carbon, inorganic zeolites, silica, or polymeric-based materials [60]. Functionalization of nanocellulose can be done depending on the type of contaminant. Typical procedures include adding carboxyl-, amino-, or thiourea-using chemical modifiers such as ethylenediaminetetraacetic acid (EDTA) or carboxymethyl groups to enhance heavy metal adsorption [61]. While applications of nanocellulose for heavy metal ion and other contaminants adsorption have been reported, the use of 3D printing to create systems such as filters and membranes for water purification remains largely unexplored.

### 3.2. Food and Packaging Applications

With the need to replace petroleum-based products, there is a demand for sustainable, biodegradable materials and packaging to increase sustainability throughout the food chain, and many exciting materials can be developed using nanotechnology products [62]. The biocompatibility and suitable shear-thinning property of nanocellulose, even when used at a low concentration, provide unique features for this material to be used for food production, packaging, and sensing applications. Nanocellulose can reinforce and stabilize printable composites, enhance the printability of nutritious foods, and improve barrier properties when used with biopolymers or synthetic polymers [63,64]. A few selected applications featuring 3D printing techniques of nanocellulose composite materials are described in this section.

**Figure 10:**
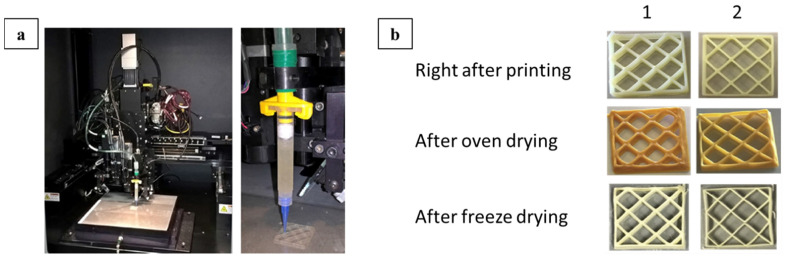
The material extrusion type of device used for 3D printing of food materials (**a**). The effect of the oven freeze-drying on the appearance of 3D-printed samples (**b**): 0.8% CNF + 50% SSMP (1) and 60% SSMP (2), used with permission from ref. [65].

Three-dimensional-printable food using CNF-based nutritional pastes, containing ingredients from all main groups of nutrients such as carbohydrates, protein, fat, and dietary fiber was reported by Martina and co-workers [65]; these pastes were formulated from mixtures of varying percentages of CNF, starch, rye bran, oat protein concentrates (OPC), fava bean protein concentrates (FBPC), skimmed milk powder (SMP), and semi-skimmed milk powder (SSMP). A simplified extrusion pump system (Figure 10) controlled by air pressure was employed in the 3D food printing through the tip of a syringe filled with the prepared and well-mixed pasted. There was a noticeable difference in the effects of the two drying methods (oven-drying and freeze-drying) used in this research on the printed samples (Figure 10), the freeze-dried samples possess higher dry matter content but less hardness compared to oven-dried samples, which makes the freeze-drying method preferable, especially for samples with initial dry matter content less than 35%. It was observed that in samples where part of the SSMP constituent was replaced with CNF, there was a reduction in the drying rate which was attributed to the high water-binding capacity of CNF and the lower dry matter content of CNF-containing samples leading to softening of such samples. The construct printed from 0.8% CNF-50% SSMP composite was achieved without clogging of printer tips, providing high printing quality and good structural stability after freeze-drying and oven-drying. High-quality printing was also achieved with pastes containing 60% SSMP due to higher yield stress and good post-printing shape stability. The study highlighted the importance of optimizing critical parameters, such as the solid content, drying rate, and hardness, that affect the composition and the shape stability of the printed paste.

**Figure 11:**
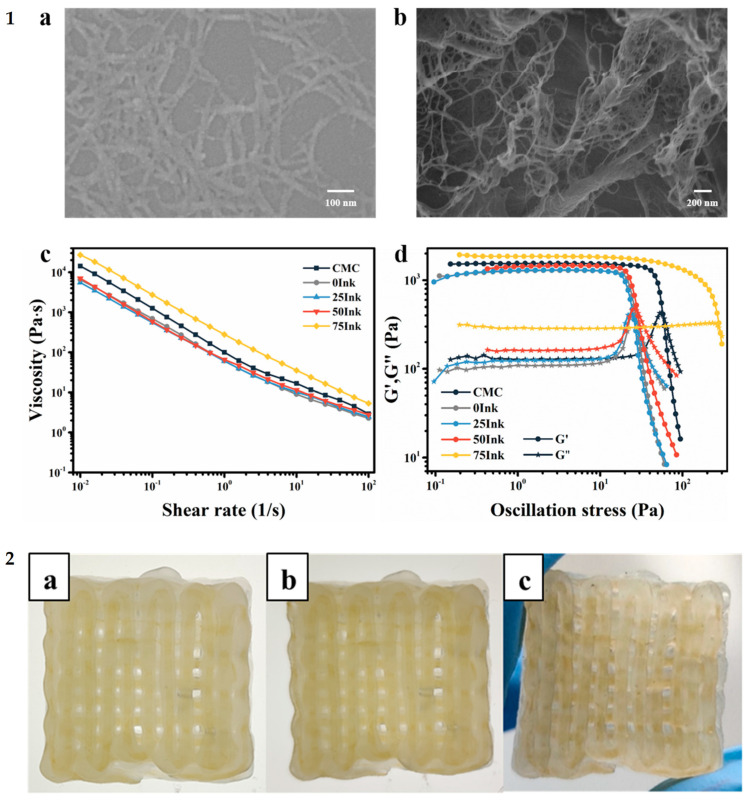
SEM images of CMC before (**1a**) and after freeze-drying (**1b**). Viscosity as a function of the shear rate for CMC and CMC-based inks (**1c**), G′ and G″ as a function of the shear stress for CMC and CMC-based inks (**1d**); Photographs of CMC-based 3D-printed aerogel before curing (**2a**), after curing (**2b**) and after freeze-drying (**2c**), used with permission from Ref. [50].

**Figure 12:**
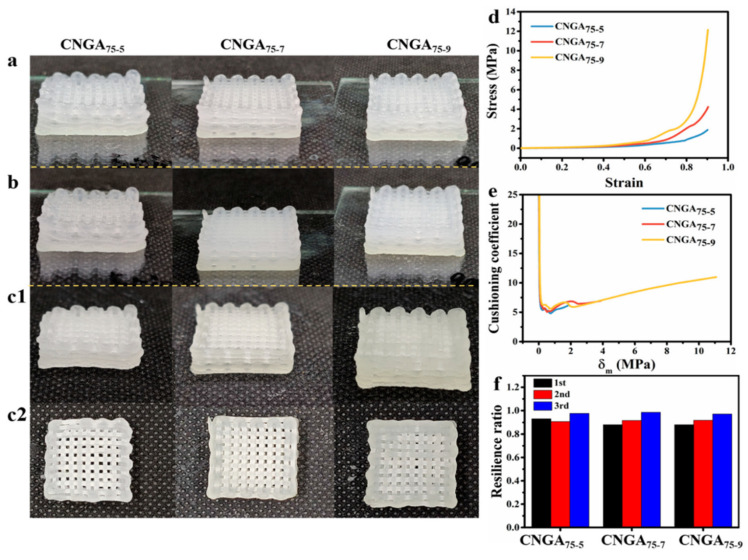
Morphology of 3D-printed samples by 75ink with different crosslinking times, before (**a**) and after (**b**) UV curing and after freeze-drying (**c1**,**c2**). (**d**) Stress-strain curves, (**e**) cushioning curves, and (**f**) thrice compression resilience ratio of samples with different crosslinking times, used with permission from ref. [50].

Alternative packaging materials are also used to preserve the quality of packaged food. As much as 30% of the loss rate of fruits was attributed to mechanical damage during transportation [66]. The irreversible loss in the quality of vegetables and fruits is due to the vibration, collision, shock, and static pressure they are subjected to during their transportation [67,68]. The difficulties in degrading and recycling the commonly used foamed polyethylene (EPE) and polystyrene (EPS) packaging materials [6] call for the development of an eco-friendly biodegradable packaging material. Spoilage of fruits and vegetables due to the actions of microbes during transportation and storage is another pressing issue in the food packaging industry. A 3D-printed food packaging material with cushioning and antimicrobial dual-function from ink based on carboxymethyl nanocellulose (CMC) was recently reported [50]. First, the fibers of CMC were discovered to form entangled 3D network structures after freeze-drying, suggesting its suitability for fabricating cushioning aerogel for packaging. To prepare the CMC-based 3D-printable ink, shell ink, Irgacure 2959, and N, N′-methylenebis(acrylamide) were mixed with 8 mL of varying concentrations (75, 50, 25, and 0%) of glycerin solution, and the mixture was heated in a water bath to aid complete dissolution. The obtained solutions were added to a mixture of CMC, Irgacure 2959, and N, N′-methylenebis(acrylamide) under constant stirring. The rheology of CMC and CMC-based inks reveals the positive impact of increasing glycerin concentration. The best printability was achieved with the composite containing the higher amount of glycerin, which had the largest yield stress and highest viscosity at the lowest shear rates (Figure 11), indicating better printability. The formulated inks were printed via coaxial 3D printing technology, cured by UV for 7 min and freeze-dried. The printed sample with 75% glycerin (CNGA_75-7_) fully retained its shape after freeze-drying, while the samples with lower glycerin concentrations had some degree of shrinkage and deformations. To study the effect of curing duration on crosslinking efficiency, CNGA_75_ samples were exposed to UV rays for 5, 7, and 9 min and then lyophilized (Figure 12**)**. In general, increase in stress under the same strain and the elastic modulus of the aerogels were observed as the crosslinking time increases, while their resilience and cushioning performance were decreasing. The CNGA_75-7_ with 7 min curing time was suggested as a good candidate for fruit packaging. Chitosan/AgNPs inside the aerogels by coaxial 3D printing technology were used to form a core–shell fiber with a translucent matrix shell and a yellow chitosan/AgNPs core. The degradation, swelling properties, cushioning performance, and silver release behavior was evaluated. The 3D-printed cushioning packaging exhibited antibacterial potency against *Escherichia coli* and *Staphylococcus aureus* [50].

**Figure 13:**
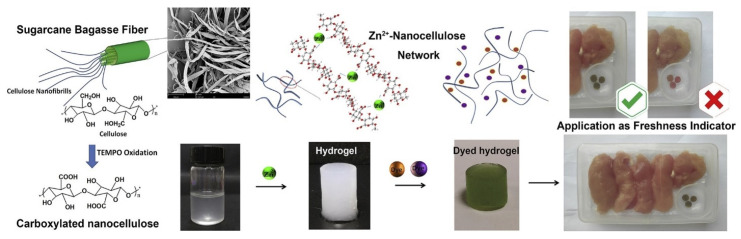
Design of CNF-based smart freshness indicator prepared by a 2,2,6,6-tetramethyl piperidinyl-1-oxyl (TEMPO) catalysis method for monitoring degradation in packaged meat, used with permission from ref. [44].

In addition to food packaging, nanocellulose hydrogels could be used to create smart indicators for intelligent packaging [62]. For example, a sugarcane-based nanocellulose hydrogel was developed in the form of a pH-responsive freshness indicator that changes color when meat deteriorates. The concept is illustrated in Figure 13, showing the formation of the Zn^2+^-nanocellulose network prepared by induced gelation of carboxylated CNFs from cellulose filaments [44]. The nanocellulose hydrogel was used as a carrier for a pH-responsive dye and as a sorbent for CO_2_ to improve the color sensitivity of the hydrogel. The indicator color was correlated with the CO_2_ and microbial growth and changes in volatile compounds as a freshness indicator in packaged chicken breast. Such smart labels made of hydrogels can be easily fabricated by 3D printing using 3D extrusion bioprinting [9], which enables printing of viscous inks of viscosity up to 6 × 107 mPa.s. The viscosity and ink composition can be easily tuned to achieve printability and mechanical properties by varying the hydrogel components’ composition, gelation conditions, and rheology characteristics. Using 3D printing, such sensors can be manufactured inexpensively and in large quantities.

### 3.3. Energy and Electrochemical Devices

Additional applications of 3D-printed nanocellulose composites have been explored in the development of energy and electrochemical devices. In the conventional approaches, conductive inks contain expensive noble metals at a concentration of about 60% to augment the conductivity of the inks, but this high concentration of metals makes the ink toxic [69] and reactive to air and other environmental factors, which reduce its chemical stability. Nanocellulose was explored as a biocompatible material to add to the ink to improve the properties of conductive inks in an effort to reduce toxicity and improve the sustainability of energy and electrochemical devices.

**Figure 14:**
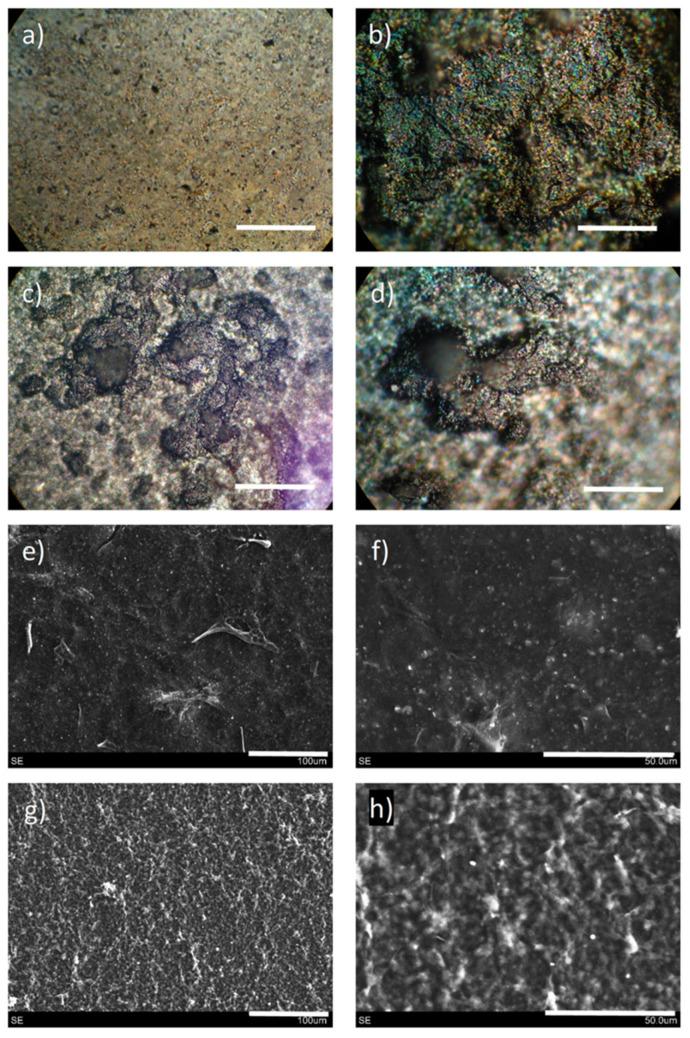
Optical photographs (**a**–**d**) and SEM images (**e**–**h**) of films obtained with low-viscosity inks (**a**,**b**,**e**,**f**) and high-viscosity pastes (**c**,**d**,**g**,**h**). Scale bars (in white) = 1 mm (**a**,**b**), 400 µm (**c**,**d**), 100 µm (**e**,**g**), and 50 µm (**f**,**h**). Each image (either optical or from SEM) corresponds to a random point of each sample, none is the direct magnification of another. Used with permission from ref. [70].

**Figure 15:**
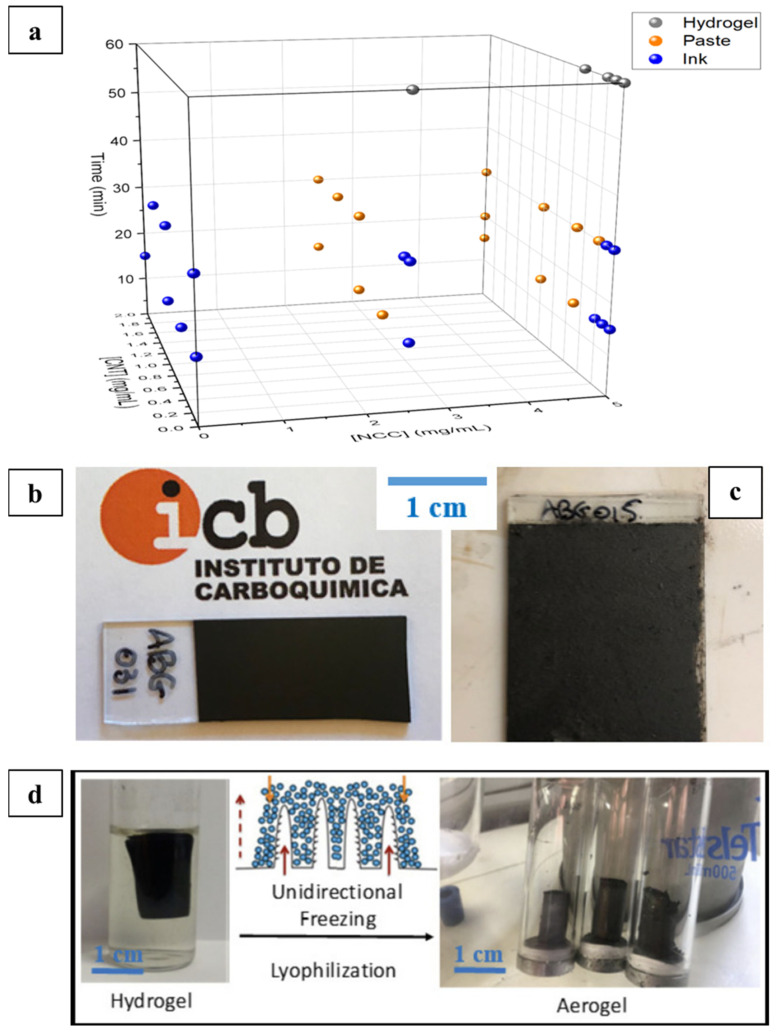
Three-dimensional scatter plot describing the effect of CNTs added in basic pH and conditions to obtain liquid inks (blue), viscous pastes (orange), and self-standing hydrogels (gray) (**a**). All samples had basic pH. Photographs of films made from low-viscosity inks (**b**) and high-viscosity pastes (**c**) on glass substrates. Real images of a hydrogel derived from hydrothermal treatment in water (**left**) and preparation scheme of aerogels by unidirectional freezing followed by lyophilization (**right**) (**d**), used with permission from ref. [70].

Films fabricated from graphene- and nanocellulose-based inks and pastes with high chemical stability and no record of cytotoxicity were developed as metal-free conductive substitutes from water-based inks with potential for applications in the production of liquid-phase electronic device constructs [70]. These films presented interesting electronic properties, suggesting potential applications as a substitute for the metal-containing conductive inks for electric or optoelectronic devices such as sensors, supercapacitors, and solar cells. Nanocrystalline cellulose (NCC), NCC type I, and NCC type II were prepared with different stirring and heating temperature conditions using an established procedure [71]. These NCCs were used as a dispersing agent for graphene oxide (GO). Varying concentrations of carbon nanotubes (CNTs) in water were tested, and aqueous ammonia (NH_4_OH) enabled a reaction medium with a basic pH suitable for the chemical crosslinking and aggregation of the graphenic nanomaterials during the hydrothermal treatment at 180 °C. The procedure provided low-viscosity inks, high-viscosity paste, or self-standing hydrogel. Conductive films were then formed by spray-coating the inks on glass substrates while the pastes were deposited via a rod-coating method on an agate rod or glass substrates. The resulting films were characterized in terms of resistivity, morphology, microstructure, thickness, and surface roughness, and it was observed that films from low-viscosity inks possessed smoother surfaces and negligibly smaller pores than the rough-surfaced films from the pastes having more prominent pores [71]. The morphology of the resulting structures, shown in Figure 14, indicates that hydrothermal treatment over 30 min always results in hydrogel formation, irrespective of the other parameters. Subjecting this hydrogel to unidirectional freezing, followed by lyophilization, leads to the formation of anisotropic porous-microstructured aerogels (Figure 15) applicable to energy and environmental remediation applications.

Table 1 summarizes examples of 3D printable nanocellulose composites, their sources, formulation, printing method and applications reported in literature.

## 4. Conclusions

Three-dimensional printing of nanocellulose-based materials has shown tremendous potential in recent years as evidenced by the increased scientific interest and research work resulting in diverse applications. Recent progress to incorporate 3D printing into industrial applications and the use of nanocellulose as a filler for various composites provides exciting new opportunities for applications due to the excellent characteristics that this material provides, such as enhanced rheological properties, sustainability, biocompatibility, mechanical strength, biodegradability, non-toxicity, and environmental friendliness. This paper discussed the different strategies to produce 3D-printable composites and structures made of nanocellulose with excellent printability and fidelity.

Although a great deal of research has demonstrated the benefits of nanocellulose, studies that explore the printing of these materials in manufacturable products are only in their infancy. Before their potential is realized, more work needs to be done to improve printability and applications. Several future directions could be envisaged. First, there is a need to improve characteristics of nanocellulose-based inks and identify complementary materials and formulations for obtaining suitable printable compositions that result in strong and mechanically stable constructs for applications. A library of tabulated viscosity and rheological properties in relation to the materials’ physicochemical properties, the type, content, and stability in the composite mixture, along with the curing conditions, would be beneficial for future developments. Second, more attention needs to be paid to the structural characteristics and understanding of the fundamental changes in the structure and properties of nanocellulose in the composite form upon printing. So far, most optimization on the printability of these composites has been done on a relatively macroscopic scale, and therefore the need for fundamental understanding of these materials that could further accelerate the development of 3D-printable materials is critical for translating this research into new products and applications. Third, the potential of these materials for applications is only beginning to be explored. Further innovations in nanocellulose modification chemistries are needed to add functionality at the surface, which could result in increased mechanical strength, barrier and sorption properties, as well as selectivity for applications such as environmental remediation and sensing. Fourth, there is a need to evaluate the environmental fate and lifecycle of these materials to fully understand their transformation and biodegradability in the environment and/or confirm their biocompatibility upon processing.

In summary, nanocellulose provides innovative solutions to social problems concerning sustainability and moves away from the reliance on petroleum-based products. The recent developments discussed in this review summarize the research status and needs necessary to advance the manufacturability and applications of these promising materials. Future efforts should concentrate on improving printability and understanding the fundamental properties of nanocellulose in printed constructs to enable advanced applications.

## Figures and Tables

**Table 1:** Examples of 3D-printed nanocellulose composites, treatment, and formulation conditions for applications.

Nanocellulose Treatment	Type and Source	Formulation	Printing Method	Area of Application	Reference
Hydrothermal and soda pretreatment, PEG plasticizer, interdigitated electrodes, relative humidity sensing	CNF from bagasse (sugarcane residue)	FS_T3.8_P40; CNF film from bagasse treated with NaOH and 3.8 molar, 40% PEG, IDE screen printing	Screen printing via flatbed screen printer (Everbright S-200HF)	Environmental (relative humidity sensing)	[56]
Carboxymethyl nanocellulose, glycerin, acrylamide, chitosan/AgNPs, cushioning, and antibacterial composite	CMC, (commercially sourced)	(CNGA/C–AgNPs); cushioning 3D-printed structure from 75 vol% glycerine, 1.2 g of N-(2-hydroxyethyl) acrylamide, and functionalized with chitosan–silver nanoparticle (Cts/AgNPS)	Coaxial printing technology via Y&D7300N 3D printer for matrix printing while LSP04-1A syringe pump extrudes the core solution (Cts/AgNPs) concurrently	Food packaging with cushioning and antimicrobial property	[50]
Fused filament fabrication (FFF)-3D printing. Sisal nanocellulose, polylatic acid composite	CNF from raw sisal fibers	(3D-PLA/1CNF); polylactic acid with varying CNF concentrations (1/3/5%)	Compression molding and 3D printing using FFF Desktop 3D printer (Fracktal Works Julia V2)	Diverse	[59]
Hydrothermal treatment, hydrogel–aerogel unidirectional freezing conversion	CNC from cotton-linters-derived microcrystalline cellulose (MCC) powder.	NH_4_OH for basic medium, carbon nanotube, and CNC for reinforcement and higher viscosity, reduced Graphene oxide (GO) for conductivity	Spray and rod coating	Metal-free electrodes (electrochemical)	[70]
Plant pulp, nutritional pastes, drying condition	CNF from birch kraft pulp	10% cold swelling starch + 15% SMP, 60% SSMP, 30% rye bran, 35% OPC or 45% FBPC	Extrusion-based printer	Food printing	[65]
